# Consumer-Led Investigation into Potential Issues That Arise When Testing Dairy Matrixes for Gluten With the NIMA Sensor

**DOI:** 10.1093/jaoacint/qsad092

**Published:** 2023-08-07

**Authors:** Tricia Thompson, Adrian Rogers, Johnna Perry

**Affiliations:** Gluten Free Watchdog, LLC, 348 Summer St, Manchester, MA 01944, United States; Bio-Check, UK, Spectrum House, Llys Edmund Prys. St Asaph Business Park, St Asaph, Denbighshire LL17 OJA, United Kingdom; In Johnna’s Kitchen, 1306 Barford Dr, Liberty, MO 64068, United States

## Abstract

**Background:**

Some consumers with celiac disease use personal, point-of-use gluten detection devices to test food. False-positive results may occur due to sampling, matrix effects, and sensor issues.

**Objective:**

The purpose of the present study was to determine if the positive gluten results some users were obtaining when testing cream cheese and materials of similar consistency were false positives and, if so, what might be causing them to occur.

**Methods:**

Cream cheese, soft cheese, and yogurt were tested for gluten using the Ridascreen Gliadin R7001 sandwich R5 ELISA and the Ridascreen Gliadin R7021 competitive R5 ELISA. Two test portions were taken, extracted, and tested from each homogenized material. Materials were also analyzed for gluten using a NIMA sensor, a personal, point-of-use gluten detection device. Multiple test portion weights were tested beginning at 0.13 to 0.17 g (the ideal weight of the test portion according to the NIMA sensor development team).

**Results:**

Using the sandwich R5 ELISA and the competitive R5 ELISA, all materials tested below the lower LOD for gluten. Using a NIMA sensor, as the weight of the test portion tested increased, sensor results went from no gluten found, to gluten found, to no test result.

**Conclusion:**

The gluten found results using the NIMA sensor are likely false positives that appear to correspond with the weight and volume of the material tested, as well as the viscosity. There is also an apparent disconnect between the gluten found result reported by the sensor and an interpretation of the lateral flow device (LFD) strip result when assessed by eye which should also be taken into account. Ideally, NIMA sensor users should be advised on the weight amount of material to analyze and test portions should be weighed before being used with the NIMA sensor. However, this is not a practical solution when testing in many environments, including restaurants.

**Highlights:**

Slight variations in weight and volume of test materials can result in false positive results when testing dairy matrixes for gluten using the Nima sensor.

Over the last few years, there has been a proliferation of analytical devices that are designed to be used by the consumer to test food themselves for the presence of gluten. This testing is undertaken outside of the confines of a traditional laboratory environment. Putting testing and sampling into the hands of the consumer brings about a series of unique challenges that, if not handled correctly, could have severe consequences, including a restricted diet for the consumer or brand damage for the manufacturer of the food.

In October 2022, Gluten Free Watchdog (GFWD), a consumer advocacy group that commissions testing of foods for gluten, began receiving emails from concerned consumers who used the NIMA sensor, a personal, point-of-use gluten detection device. Various cream cheeses and other materials of similar consistency were periodically testing “gluten found”. The NIMA sensor is a combination lateral flow device (LFD), and electronic sensor with an algorithm that interprets the results of the test strip (1). Results are reported as either a smile icon (meaning no gluten detected), a wheat symbol with the words “gluten found,” an exclamation point with the words “no test result,” or an exclamation point with the words, “capsule error” (2).

Consumers have reported using the NIMA sensor for any number of reasons, but one statement that is made repeatedly on GFWD social media platforms is that even though the sensor may not be perfect, it is better than nothing. Consumers have also stated that testing using a NIMA sensor is their only recourse to determine if a product contains gluten. Users have also reported that the sensor has saved them from gluten cross-contact due to a gluten found result. The assumption on the part of the consumer is that the result is accurate (3).

GFWD was asked by consumers who had either tested the cream cheese materials themselves or had seen the results posted on social media to assess some of the NIMA sensor-tested materials using an ELISA. Materials that were giving inconsistent results with the NIMA sensor when used by consumers and reported to GFWD in October 2022 gave a negative result for gluten when tested in the laboratory using ELISAs in November 2022. Study authors have previously reported that consumer testing of food with LFDs may sometimes lead to false-positive results (4). This may be due to several factors, including the amount of food sampled for testing and resulting changes in pH levels (4). The purpose of the present study was to determine what was causing the gluten found results and why results using the NIMA sensor were different when different users tested similar materials.

## Experimental

### Preparation and Handling of Materials for ELISA and NIMA Sensor Testing

Products were purchased for testing by either GFWD or our consumer tester (i.e., co-author Johnna Perry). The ELISA testing undertaken at the third-party laboratory was carried out prior to testing using the NIMA sensor. Hence, test materials analysed at the third-party laboratory versus those tested using the NIMA sensor were from different packages of product. Testing by the consumer using the NIMA sensor was undertaken on four separate dates. Testing by our co-author was undertaken in the same manner as a consumer would test and involved no homogenization of the test portions. Replicate testing with the NIMA sensor was limited due to availability of NIMA sensor test capsules. All ELISA testing at the third-party laboratory was carried out on the same date following the kit manufacturer’s instructions regarding amount of representative material to homogenize and test portion size.

### Testing Using ELISAs

On November 15, 2022, GFWD commissioned gluten testing on Philadelphia Cream Cheese original block, Philadelphia Cream Cheese Spread (strawberry flavor), Boursin Cheese Garlic & Herbs, and Noosa Lemon Yoghurt. Only the yoghurt was labeled gluten-free. The other materials appeared to be free of gluten-containing ingredients. Representative test portions were analyzed for gluten at Bia Diagnostics, LLC, Colchester, VT, United States (ISO Accredited Lab) using the Ridascreen Gliadin R7001 sandwich R5 ELISA and extracted with the cocktail solution (Art. No. R7006) following the kit manufacturer's instructions (R-Biopharm, Darmstadt, Germany; 5). Test portions were also analyzed using the Ridascreen Gliadin R7021 competitive R5 ELISA and extracted with ethanol following the kit manufacturer’s instructions (6). Two test portions were taken, extracted, and tested from each homogenized material.

### Testing Using the NIMA Sensor

Testing using the NIMA sensor was undertaken by our consumer tester (i.e., co-author Johnna Perry) using her personal device. She was given very little direction except that she should start testing portions around 0.15 g and gradually increase the test portion weight. She was asked to take photos of all food portions tested, their weights on the balance, the results on the NIMA sensor (smile, gluten found, no test result), and the visual results on the test strip (i.e., for the presence/absence of test line and control line; if no control line was observed then the test was deemed to be invalid).

Testing using the NIMA sensor was undertaken on November 22, 2022, November 30, 2022, Jan 7, 2023, and June 7, 2023, at either a college laboratory (William Jewell College Bio Lab 137) weighing test portions using a Denver Instrument SI-234 balance, or in a gluten-free environment in the consumer’s home weighing test portions using Weigh Gram Top 100 scale (to mimic how a consumer would test at home using a kitchen scale). For testing undertaken at the college laboratory in November, 2022 (but conducted by the consumer), single test portions (not homogenized) of Philadelphia Cream Cheese original block, Philadelphia Cream Cheese Spread (strawberry flavor), Boursin Cheese Garlic & Herbs, and Noosa Lemon Yoghurt were analyzed at test portion weights from 0.13 to 0.17 g (Table 1). These starting weights were chosen as a direct response to previous correspondence with the NIMA sensor development team who stated the ideal weight of the test portion to be analyzed with the NIMA sensor is 0.15 ± 0.02 g (personal communication, nameredacted@nimasensor.com, January 11, 2017).

**Table 1.** NIMA sensor results^a^

Material	Test portion, g	Sensor reading	Visual observation of test strip
Boursin Cheese Garlic & Herbs	0.16	Smile^b^	No test line
Noosa Lemon Yoghurt	0.16	Smile	No test line
Noosa Lemon Yoghurt	0.35	Smile	No Test Line
Philadelphia Cream Cheese Spread (Strawberry Flavor)^c^	0.17	Smile	No test line
Philadelphia Cream Cheese Original Block^d^	0.16	Smile	No test line
Control (Dunkin Donut hole)	0.16	Gluten found	Test line

a Testing undertaken in a college laboratory using a Denver Instrument SI-234 balance on November 22, 2022.

b Smile = No gluten detected.

c Best by date March 7, 2023.

d Best by date March 18, 2023.

A 0.35 g test portion of yoghurt (approximating the weight of a pea was also tested; Table 1). The NIMA Partners website states that the amount tested should be approximately the size of a pea (1). A pea was weighed by the consumer at approximately 0.39 g.

Additional testing of the cream cheese block and cream cheese spread using a NIMA sensor was undertaken at the consumer’s home on November 30, 2022, and January 7, 2023. Test portions were weighed using a Weigh Gram Top 100 scale. Test portions were tested from around the weight of a pea up to approximately 1.5 g (Table 2).

**Table 2.** NIMA sensor results^a^

Material	Test portion, g	Sensor reading	Visual observation of test strip
Philadelphia Cream Cheese Original Block (Testing dates 11/30/22, 1/7/23)^b^			
Test date: 11/30/22; Best by: 1/16/23	0.46	Smile^c^	No test line
Test date: 11/30/22; Best by 1/16/23	0.80	Smile	No test line
Test date: 1/7/23; Best by 4/3/23	0.95	Smile	No test line
Test date: 1/7/23; Best by 4/3/23	1.06	Smile	No test line
Test date: 1/7/23; Best by 4/3/23	1.08	Gluten found	No test line
Test date: 1/7/23; Best by 4/3/23	1.12	No test result	No test line
Test date: 11/30; Best by 1/16/23	1.17	No test result	Invalid test
Test date: 11/30; Best by 1/16/23	1.61	No test result	Invalid test
Philadelphia Cream Cheese Spread (Strawberry Flavor) (Test date 1/7/23)^d^			
Test date: 1/7/23	0.38	Smile	No Test Line
Test date: 1/7/23	0.52	Gluten found	Test line present
Test date: 1/7/23	0.66	No test result	Invalid test
Test date: 1/7/23	0.96	No test result	Invalid test
Test date: 1/7/23	1.21	No test result	Invalid test
Test date: 1/7/23	1.52	No test result	Invalid test

a Testing undertaken in a gluten-free environment using a Weigh Gram Top 100 scale on November 30, 2022, and January 7, 2023.

b Best by dates January 16, 2023, and April 3, 2023.

c Smile = No gluten detected.

d Best by date March 14, 2023.

Testing of Philadelphia Cream Cheese Original block was repeated on a separate fourth occasion (Table 3) on June 7, 2023, using the NIMA sensor to demonstrate the repeatability of the testing.

**Table 3.** NIMA sensor results for Philadelphia Cream Cheese original block^a^

Test portion, g	Sensor reading	Visual observation of test strip
1.02 (sample 1)^b^	Smile^c^	No test line
1.04 (sample 1)	No test result	No test line
1.04 (sample 2)	No test result	No test line
1.06 (sample 1)	Smile	No test line
1.06 (sample 2)	Gluten found	No test line
1.06 (sample 3)	No test result	No test line
1.08 (sample 1)	No test result	No test line
1.08 (sample 2)	No test result	No test line
1.12 (sample 1)	No test result	No test line
1.12 (sample 2)	Gluten found	No test line
1.12 (sample 3)	No test result	No test line

a Testing undertaken in a gluten-free environment using a Weigh Gram Top 100 scale on June 7, 2023.

b Best by date September 16, 2023.

c Smile = No gluten detected.

Because analysis of test portions using the NIMA sensor took place on four separate dates, multiple packages of products were tested. Four blocks (with different best by dates) of cream cheese were tested. Two tubs (with different best by dates) of cream cheese spread were tested. Different best by dates of the cream cheese block and cream cheese spread were tested by the consumer not by design but based on availability.

## Results

### ELISA Results

Using the sandwich R5 ELISA (used to assess materials for intact gluten protein), all materials tested below the lower LOQ of 5 mg/kg gluten. All materials also tested below the LOD of 1 mg/kg of gluten (mean value, matrix-dependent; 5).

Using the competitive R5 ELISA (used to assess materials for gluten protein fragments), all materials tested below the lower LOQ of 10 mg/kg gluten. All materials also tested below the LOD of 4.6 mg/kg of gluten (mean value, matrix-dependent; 6).

### NIMA Sensor Results

As the weight of the test portion analyzed increased, sensor readings went from smile, to gluten found, to no test result (Tables 1–3). There is also an apparent disconnect between the gluten found result reported by the sensor and an interpretation of the LFD strip result when assessed by eye.

## Discussion

### False Positives

The gluten found results using the NIMA sensor are likely false positives. [Due to the apparent disconnect between the sensor result and visual assessment of the strip, the authors are hesitant to label the results of the cream cheese analysis with the NIMA sensor as cross-reactivity (Tables 2 and 3).] When the materials were tested using the sandwich and competitive R5 ELISAs, the level of gluten was below the lower LOD for both assays. All four materials received a smile result when the “right” weight amount was tested using a NIMA sensor (Table 1). Two materials (i.e., yogurt and cream cheese spread) were tested at the approximate weight of a pea and they also received a smile result (Tables 1 and 2). Consumers are advised on the NIMA Partners website to test a pea-sized amount. To the best of the authors’ knowledge, the approximate weight of a pea (0.3 g according to the manual) is provided only in the NIMA sensor manual (2) and then only under the entry for testing brightly colored foods. It is not provided in the NIMA sensor “Quick Start Guide” (7) or on the page “How it Works” (1).

### Potential Impact of Test Portion Weight on Results

The results using a NIMA sensor suggest that as the test portion weight increases the test findings may become increasingly unreliable. Two materials (i.e., cream cheese block and cream cheese spread) were tested using a NIMA sensor by our consumer. As is illustrated in Table 2, as the weight amount of the test portion increased, results moved from a smile to gluten found to no test result. According to the NIMA Partners online manual (2), an exclamation point, with the words “no test result” means the sensor is unable to provide a conclusive result due to test portion size, food type, chemistry, or a mechanical issue (2). In the case of the original cream cheese block material, the addition of 0.02 g was the difference between the sensor giving an indication that the food was safe to eat and giving an indication that the food was not safe to eat. An additional 0.04 g prevented the material from running at all. Other research groups have also reported that depending on the material tested, test portion weights sometimes impact the sensor results, especially when the material is viscous (8).

### Potential Impact of Volume on Results

**Figure 1.**
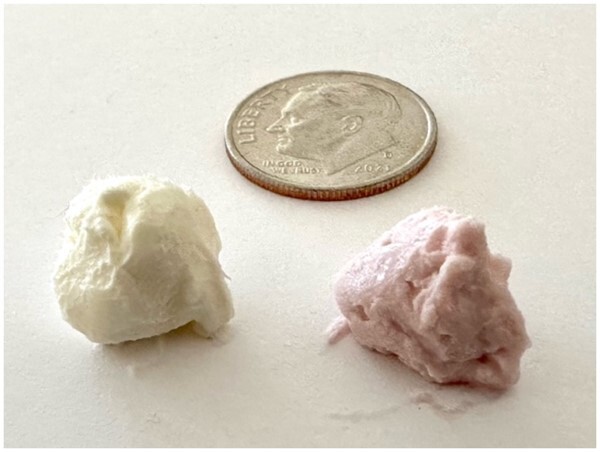
Cream cheese original block weighing 1.08 g and strawberry cream cheese spread weighing 0.52 g. Photo credit: Johnna Perry.

In addition to weight, the volume tested also may be an issue. Strawberry cream cheese spread tested gluten found at a weight of 0.52 g, while the cream cheese original block tested gluten found at a weight of 1.08 g (Table 2). Both materials appear to be approximately the same volume regardless of weight (Figure 1).

### Potential Issue of Flow on Test Results

The false positives may be due to flow issues. NIMA sensor users are provided with slightly different guidelines to address foods that are thick or dense. The NIMA Partners gluten manual (2) states that such materials should be tested with a 1:1 dilution of water “to prevent clogging and slow flow” and that if this is not done, the sensor may give a false-negative result. However, best practices on the NIMA Partners webpage (9) state that a smaller amount should be used. The findings of the present study indicate that the viscous materials tested (e.g., cream cheese) may yield false-positive results. According to Kainz et al. (10), the different viscosities of different materials is one of the challenges of lateral flow tests. The viscosity of a material may result in either underestimating or overestimating the analyte (e.g., gluten) being tested for depending upon how fast the material flows through the test strip.

In the manufacturer validation of the sensor (11), the authors note that fat-containing foods “can clog pores and hinder capillary flow” in LFDs. The authors of the validation study note that in their tests with the NIMA sensor the test strips ran properly at medium weights of salad dressing, yogurt, chocolate, butter, and cheese, but that some larger amounts clogged the test strips. In their tests, the result was an error reading. Pea-sized amounts (∼400 mg) received correct gluten-free readings.

### Potential Impact of the NIMA Sensor and Algorithm on Results

**Figure 2.**
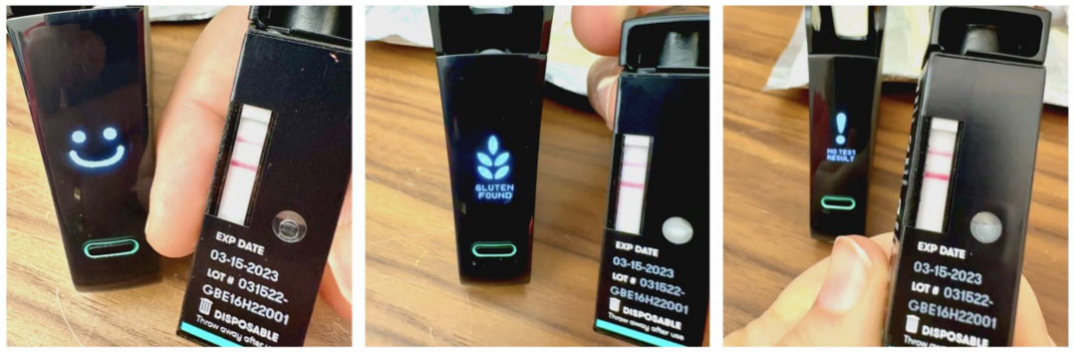
LFD test strips and corresponding NIMA sensor readings. Photo credit: Johnna Perry.

There may also be issues with how the NIMA sensor reads the LFD test results. According to the NIMA Partners website, both an electronic sensor and algorithm detect the test result (1). The website also states that the sensor reading should be deferred to over the reading of the LFD test strip using the naked eye (1). However, in the manufacturer validation of the sensor, it appears that visual readings of the test strips were used to validate the sensor readings (i.e., smile, gluten found; 11). Figure 2 includes photos of LFD test strips and the corresponding NIMA sensor readings. All three LFD test strips look very similar.

There is an apparent disconnect between the “gluten found” result reported by the NIMA sensor and an interpretation of the LFD strip result when assessed by eye which should also be taken into account (Tables 2 and 3). With the experimental work undertaken for this study, when the NIMA sensor displays a gluten found result with the cream cheese materials tested, visual interpretation of the result by eye could only be confirmed in a single instance (Table 2, Philadelphia Strawberry Cream Cheese Spread, 0.52 g test portion). In all other instances when the NIMA sensor recorded a gluten found result this could not be confirmed by visual inspection of the strip. This observation seems to be contrary to the published NIMA sensor validation information (11) whereby a visual assessment of the lateral flow strip within the NIMA sensor test capsule was used as an indication that the NIMA sensor was operating correctly.

## Conclusions

Based on testing undertaken by GFWD using the sandwich and competitive R5 ELISAs, cream cheese and various other materials that gave a gluten found result when using the NIMA sensor contain no detectable gluten. Unfortunately, some NIMA sensor users may have stopped eating certain products based on test results. While it is exceedingly important that NIMA sensor users follow directions and test only a pea-sized amount of material, consumer perception of what this amount looks like may vary. This may impact test results. It also is the case that pea-sized visual amounts of foods will have different weights. This also may impact test results. Viscosity should also be taken into consideration when assessing the ideal weight amount of test portions to use with the NIMA sensor and any LFD as it may influence the result of the test. Ideally, NIMA sensor users should be advised on the weight amount of material to test and materials should be weighed before testing. However, this is not a practical solution when testing in many environments, including restaurants. Regardless, the manufacturer of the NIMA sensor should provide very clear user instructions such as those specified in the AOAC INTERNATIONAL “Stakeholders’ Guidance Document for Consumer Analytical Devices with a Focus on Gluten and Food Allergens” (12). It also appears that some adjustments should be made so that the sensor reading is in line with the test strip findings.

This short communication was produced as a direct response to consumer questions with regard to the reliability of the NIMA sensor when testing cream cheese. Work was undertaken based on a consumer-led approach and as such the authors recognize the limitations of such a study due to the relatively small data set and limited use of replicates as a consequence of the cost and availability of NIMA sensor test capsules. The authors recommend that a further study should be undertaken to experimentally determine the observations highlighted in this short communication, namely the impact of sample weight on test results and the apparent disconnect between sensor results and the results observed by visual inspection of the lateral flow test strip.
